# Sheep Post-Domestication Expansion in the Context of Mitochondrial and Y Chromosome Haplogroups and Haplotypes

**DOI:** 10.3390/genes13040613

**Published:** 2022-03-29

**Authors:** Karolína Machová, Anežka Málková, Luboš Vostrý

**Affiliations:** 1 Department of Genetics and Breeding, Faculty of Agrobiology, Food and Natural Resources, Czech University of Life Sciences Prague, Kamýcká 129, 165 00 Prague, Czech Republic; vostry@af.czu.cz; 2 Department of Animal Science, Faculty of Agrobiology, Food and Natural Resources, Czech University of Life Sciences Prague, 165 00 Prague, Czech Republic; malkovaa@af.czu.cz

**Keywords:** domestication, mitochondrial haplogroups, matrilineal inheritance, patrilineal inheritance, Y chromosome haplotypes

## Abstract

Mitochondrial DNA and nonrecombinant parts of Y-chromosome DNA are a great tool for looking at a species’ past. They are inherited for generations almost unaffected because they do not participate in recombination; thus, the time of occurrence of each mutation can be estimated based on the average mutation rate. Thanks to this, male and female haplogroups guide confirming events in the distant past (potential centers of domestication, settlement of areas, trade connections) as well as in modern breeding (crossbreeding, confirmation of paternity). This research focuses mainly on the development of domestic sheep and its post-domestication expansion, which has occurred through human trade from one continent to another. So far, five mitochondrial and five Y-chromosome haplogroups and dozens of their haplotypes have been detected in domestic sheep through studies worldwide. Mitochondrial DNA variability is more or less correlated with distance from the domestication center, but variability on the recombinant region of the Y chromosome is not. According to available data, central China shows the highest variability of male haplogroups and haplotypes.

## 1. Introduction

Domestic sheep (*Ovis orientalis* Linnaeus, 1758), together with domestic goat (*Capra aegagrus hircus* Linnaeus, 1758), were among the first livestock to be domesticated through several domestication events between the eleventh and eighth millennia BP [1,2,3]. Throughout almost ten millennia, domestic sheep have spread with the help of man to almost all continents, different climatic zones, and altitudes. This adaptability and production variability are naturally rooted in its genome. Indeed, most of this functional genetic diversity comes from wild ancestors in which they have already been segregated [4,5]. Scientists are increasingly seeking functional genes that cause this natural adaptability due to their potential use in marker-assisted selection [6,7,8].

The urial (*Ovis vignei* Blyth, 1841) was first considered the main ancestor of domestic sheep, and only after the number of chromosomes in individual related species of the genus *Ovis* was revealed, the scientific public leaned towards the theory of a single ancestor, the European mouflon (*Ovis orientalis musimon* Pallas, 1811) [9]. According to the latest findings supported by several mtDNA haplotype studies [10,11,12,13], the direct ancestor of the domestic sheep appears to be the Asian mouflon (*Ovis gmelinii* Gmelin, 1774), while a close relative of the sheep, the European mouflon, appears only to be a feralized remnant of the originally domesticated sheep. This statement was confirmed when the same retrotypes were found in the Corsican, Cypriot, and Sardinian mouflons as in primitive Nordic sheep breeds [14]. Modern breeds are characterized by a high frequency and fixation of the retrotype called *enJSRV-18*. In contrast, primitive populations, including the European mouflon, do not carry this retrotype. Instead, they either have a high frequency of *enJSRV-7* or are generally deficient in insertional polymorphic *enJSRVs*, including *enJSRV-7* [14].

Molecular genetic methods provide information today on the dispersion and genetic diversity of domestic sheep. Over the last few decades, significant progress has been made in the genomic sequencing of animals, including sheep [15]. However, many methods targeting specific sections of the genome are also used to study the genetic diversity of sheep: the study of haplotypes (mitochondrial and non-recombinant parts of the Y chromosome), autosomal microsatellite markers, and, most recently, single nucleotide polymorphisms, SNPs [16]. With the increasing amount of genetic information available, our information on the origin, development, adaptation mechanisms, and variability of livestock is becoming more accurate [15]. For the study of development and origin, information from Y or mt haplotypes of primitive national breeds, or even better directly from archaeological finds, is particularly valuable [17]. Indigenous breeds are not expected to have a larger proportion of newly introduced genes, as their development is closely linked to the development of ethnic groups, which usually still breed them in the traditional pastoral way in certain areas for many centuries and millennia [18,19,20].

Some studies even point to the possibility of using recombinant sections of gonosomes. Diversity on the X chromosome and autosomal chromosomes in wild and domestic sheep across continents revealed a decrease in the diversity of single nucleotide polymorphisms (SNPs) on the X chromosome compared to autosomes [21]. On the other hand, a smaller number of selective SNPs are found on the X chromosome, probably because most target loci and genes that are long-term are affected by human selection and are located on autosomes. Chessa et al. [22] focused on these loci and demonstrated that even in the functional regions of the sheep genome, there is considerable genetic variability, reflecting years of adaptation, natural or artificial selection, migration, and crossing. They can, therefore, also be used to study current biodiversity.

This review aims to summarize the current knowledge about the colonization dispersion of domestic sheep based on the two most used approaches to this issue, the study of the variability of mitochondrial and nonrecombinant Y DNA. The meta-analysis in the form of graphical outputs focused on recent local and transboundary breeds. Commercial or improved breeds (e.g., Texel) were not included in the dataset.

## 2. Mitochondrial Haplogroups and Haplotypes

MtDNA is inherited through the maternal lineage and, thus, lacks recombination. At the same time, it mutates five to ten times faster than nuclear DNA [23]. This may be due to a lack of repair mechanisms or the formation of free radicals during the phosphorylation process [24]. The hypervariable region of the mtDNA regulatory region is one of the most available and effective markers for population genetic studies, which allows monitoring of the maternal lineage of the gene pool and the related phylogenetic relationships, structure, and diversity of the population [25].

Assuming that humans take only part of the animal population from the original domestication center when colonizing new areas, mtDNA haplotype studies should logically reflect the geographical progression of the domestication of sheep. Thus, the greatest diversity of mitochondrial haplotypes in sheep can be expected in the Eastern Mediterranean [26]. For autosomal diversity or diversity on the X chromosome, no higher values in the area of domestication were confirmed. Certainly, the recent breeds kept in these areas no longer represent the genotype of the original thin-tailed sheep, which were one of the first to spread further around the world [21].

Of the specific regions monitored in sheep mtDNA, researchers most often focus on the D-loop region and the cytochrome-b-coding region [27]. More than 900 haplotypes have been found for cytochrome b [28]. It is currently assumed that there are up to six different haplogroups into which they can be divided, called A, B, C, D, E, and X. However, haplogroup X has so far only been described by a single study [29], and no subsequent studies have confirmed its existence. Based on genetic material from archaeological finds, it is assumed that there were originally more haplogroups and that some of them became extinct [30]. A and B are the most common groups in sheep from Europe (B) and Asia (A) and were also the first to be identified [10,31]. C is more genetically variable than the previous two groups but has nothing in common with any wild-type sheep. To a small extent, this type occurs in native Portuguese sheep, as well as in the Caucasus, the Middle East, and Asia. The D and E haplogroups are two of the rarest and were found in the North Caucasus region [26]. D also appears to be the haplogroup closest to the common ancestor of sheep and mouflon [32]. The last haplogroup was found at the Anatolian mouflon (*Ovis gmelini anatolica* Valenciennes, 1856), and it is very close to groups E and C [29]. The period of formation of these haplogroups is assumed to be sometime in the period of 5–35 thousand years ago, which is more than 150 thousand years later than the expected separation of the Cypriot mouflon (*Ovis gmelini ophion* Blyth, 1841) [13,32].

**Figure 1:**
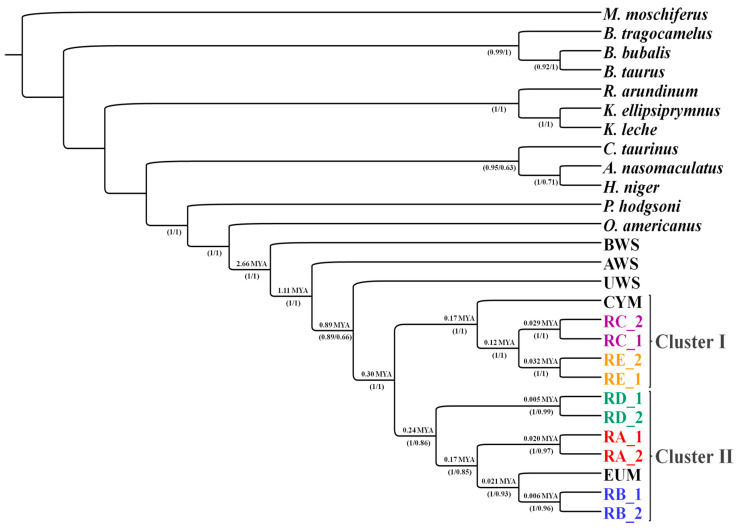
Root phylogram obtained by Bayesian inference from 28 haplogroups. The labels below the nodes indicate bootstrap values for maximum similarity. Above the nodes are the molecular datings in millions of years. Cluster 1 contains three groups, one with haplogroup E (RE), the other with haplogroup C (RC), and the third with all Cypriot mouflons (CYM) and some Anatolian mouflons. The second cluster has a total of four groups. Three haplogroups of domestic sheep were divided according to haplotypes D, A, B (RD, RA, RB), and European mouflon (EUM). Some Anatolian mouflons are also included in haplogroup A. BWS, AWS, UWS means *Ovis canadensis* (bighorn sheep), *Ovis vignei* (urial), and *Ovis ammon* (argali), respectively. Taken from [32].

In addition to the study of population dispersion, the mitochondrial genome is also used for phylogenetic analyses of the genus *Ovis*. For example, according to a study that analyzed mitochondrial cytochrome b sequences [13], argali (*Ovis ammon* (Linnaeus, 1758)) was the first of the genus *Ovis* to diverge in Europe. The Meadows collective came to a somewhat different dating based on a study of complete mitogenomes in domestic and wild sheep [33]. They determined a calibration point based on the sequence of cytochrome b of an already extinct relative of the genus *Myotragus,* which separated 5.35 mya (million years ago) [33]. Based on this, they estimated the cleavage of the two major mitochondrial haplogroups, A and B, from the unfrequented C and E to 0.92 mya, and the separation of C from E to 0.26 mya. Sanna’s team reached a different estimate of the diversification of mitochondrial haplogroups on samples of whole mtDNA sequences (see Figure 1) [32]. The first separation of the two major branches of haplogroups (C, E and A, B, D) happened 0.3 mya, according to Sanna’s team. Haplogroup D (0.24 mya) was the first to be separated, groups A and B (0.17 mya) were further distinguished, and C and E (0.12 mya) were the last [32]. Table 1 provides a comparison of the estimates of five different studies of the three main divergence points preceding the formation of individual mitochondrial haplogroups.

In any case, it is not possible to assume a connection between the divergence of any of the five haplogroups and the post-domestication expansion, which according to archaeological findings, dates as far back as the eighth millennium BP [3,36]. Based on molecular genetic data, this may have happened two millennia earlier [14,35]. For lineage B, a primary haplotype may have already been identified in 2019, when a lineage B haplotype was found in the Sardinian mouflon and which was estimated to split about 110,000 years ago, about 30,000 years earlier than the expected division of the European mouflon from sheep lineage B [37].

The sheep probably got to North America by migrating from Asia across the Bering Strait. Bighorn sheep (*Ovis canadensis* (Shaw, 1804)) and Alaskan sheep (*Ovis dalli* (Nelson, 1884)) are monophyletic from the Siberian snow sheep (*Ovis nivicola* (Eschscholtz, 1829)) from which they separated about 1.6 million years ago [13]. Similar conclusions were reached in later studies [32,38].

### 2.1. Europe

**Figure 2:**
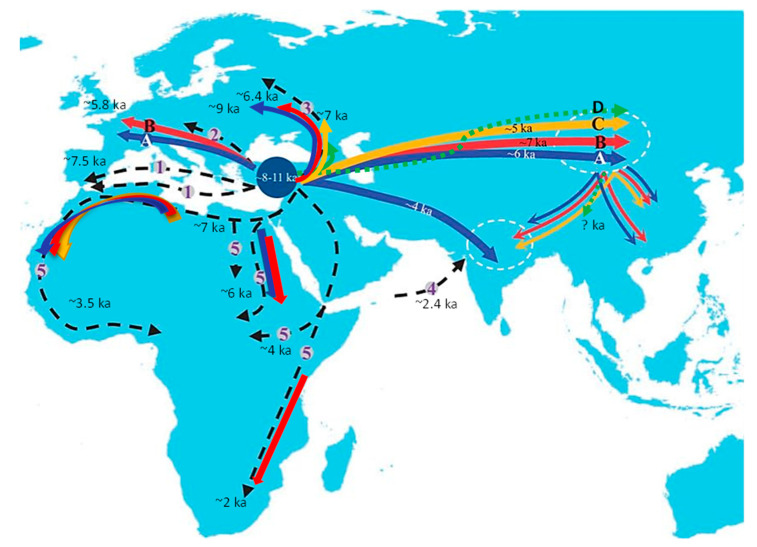
The main dispersal routes of sheep from the place of domestication over Eurasia and Africa estimated in thousands of years BP. **A**, **B**, **C**, **D** = routes of major mitochondrial lines [34,44]; 1 = Mediterranean route [1,9]; 2 = Danubian route [1,9]; 3 = route to northern Europe [45]; 4 = routes of ancient sea transport to the Indian subcontinent [46]; 5 = African routes [47,48,49,50]. Taken and modified from [34].

**Figure 3:**
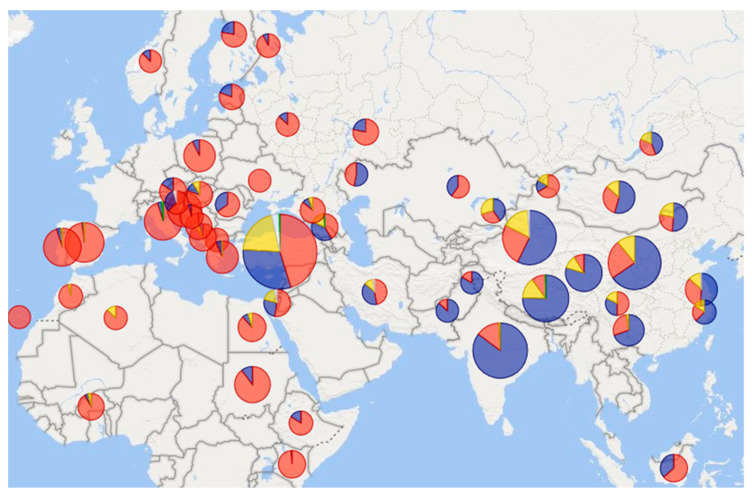
Types and frequencies of mitochondrial haplogroups in different regions of the Eastern Hemisphere. Color resolution of haplogroups: **A**, **B**, **C**, **D**, E. The data used to create this map diagram with Power BI are a compilation of data from studies: [12,17,18,19,20,25,26,29,34,42,43,44,45,46,48,49,50,51,52,53,54,55,56,57,58,59,60,61,62,63,64,65,66,67,68,69,70,71,72,73,74,75,76]. On a scale of 21–866 samples per pie chart.

The high diversity of sheep mitochondrial lineages could be due to domestication from several developmentally related ancestors [32]. For this reason, the complete dominance of haplogroup B mtDNA in Europe (Figure 2) indicates the expansion of the European population from only a few individuals. Such a low diversity was already present in herds in the Black Sea area before the Neolithic expansion [39]. With the Neolithic expansion, progress through Europe accelerated. Sheep reached central Anatolia about 10,000 years ago [1]. From there, two main dispersal routes led to Europe, via the Mediterranean Sea and through the Danubian valley. These two European pathways were also confirmed by research of small ruminant lentiviruses (SRLVs) [40]. The Mediterranean route led from Cyprus through the Balkan Peninsula and the Apennine Peninsula to Corsica and Sardinia from which northern Italy and southern France were further inhabited. Domesticated sheep reached the Iberian Peninsula around 7500 BP. The Danube road led through river valleys to Central Europe [1]. Sheep entered the Alps, either way, more than 5000 years ago [41]. However, there was probably another dispersal route to Europe, which led through Caucasus, Russia to northern Europe [11]. Lineage B was probably the first to reach Finland, followed by lineage A in the early Middle Ages [42]. Lineage A has spread across Europe through wool-refining efforts, but the origin of lineages C and D in Central Europe remains unclear. They could have come to Europe with a prehistoric man or much later, for example, during the Ottoman expansion [43]. This is consistent with the current findings of these two lineages, mainly in the Balkans and the Iberian Peninsula (Figure 3), which has been under Arab rule for almost seven centuries.

### 2.2. Asia

The colonization of Asia was a little more complicated (Figure 2). The study evaluated the optimal model based on the ABC analysis of mitochondrial lineages, which most likely occurred during the colonization of Asia, and revealed the next three steps [34]:

1. Lineage A spread first to the Mongolian Plateau and the Indian subcontinent. Later, it expanded from the Mongolian Plateau to northern and southwestern China. According to [77], lineage A was the most abundant lineage in ancient Bronze Age China (95.5%). Its abundance increased from west to east.
2. Lineage B headed first on the Mongolian Plateau and colonized northern and southwestern China and India from there.
3. Lineage C also first colonized the Mongolian Plateau. From there it headed to northern China and then to the Indian subcontinent.

The fourth lineage, D, was also found in Central Asia, specifically in the south of the Tibetan Plateau in one of the local breeds, Linzhou [44]. However, currently there are no relevant estimates of the time or route of its arrival at this location.

The mainland route to Asia, however, may not have been the only one. Another possibility of importing the maternal lineages A and B to India seems to be the sea route from the ancient port of Lóthal at the mouth of the Indus river [46]. At the time of Harappan culture (~2.4 thousand BP), this place had trade links with Africa and West Asia. The Mongolian Plateau was identified as the area with the highest variability [34]. Even several cases of heteroplasmy were found there, which must have existed in this area for several millennia [51]. The Mongolian Plateau acted as a migratory hub from which the lineages spread from the Middle East to Asia [34,52]. Specifically for lineages A and B, high nucleotide diversity is found in India [19,34] and for lineage C in northern China [34]. According to some authors, this diversity is so significant that it cannot come from the same domesticated animals that gave rise to these lineages in the more eastern areas. Therefore, independent domestication events could also have occurred on the Indian subcontinent [46] or in China [53,78].

So far, no study has confirmed that the Indonesian region has any original breed of sheep. The current breeds are, therefore, mainly descendants of European breeds imported by the Dutch in the second half of the 19th century [54] or fat-tailed sheep brought by Arab traders in the early 18th century [79]. However, these imported breeds were often crossed with local thin-tailed sheep of unknown origin, which were imported even earlier, probably by traders from Asia. [54]. This is probably the source of haplogroup A in Indonesia.

### 2.3. Africa

Evidence of the presence of sheep in Africa dates back to a much earlier time than in Southeast Asia [47]. Sheep probably penetrated North Africa through two routes about 7000 years ago. The first is the same colonization dispersion that crossed the Mediterranean Basin, the second led across Sinai, then down to and over the Red Sea [1]. There were several scattering routes on the African continent itself, south to the Middle Nile Valley, west to central Sahara, and north to Libya. Another possibility remains the spread of sheep from the Mediterranean along the northern shores of Africa. The last route discussed in Africa is the direct trade link between East Africa and the Arabian Peninsula [47]. As in Europe, mitochondrial haplogroup B is dominant in Africa (Figure 3), as confirmed in different locations—South Africa [80], Sudan [48], Kenya [49], West Africa, and the Canary Islands [50].

### 2.4. America and Australia

The settlement of the other two continents of America and Australia is already a matter of modern history. The first sheep brought to Central America by the Spaniards were either hair type (West African furry sheep) or coarse wool type (Churro breed from Iberia), which were later crossed with merino and gave rise to the Creole type of sheep [9]. However, it is highly probable that other breeds from the area of the Iberian Peninsula, such as Manchega, Latxa, Castellana, or Rasa Aragonesa, also contributed to the creation of Creole sheep [81].

The first hairy sheep were brought to America from the Canary Islands by Columbus and the first colonists and later along with slaves from other parts of West Africa [82]. West African sheep arrived in America in the early seventeenth century, and their contribution to the gene pool of contemporary hairy American sheep is the most significant [83]. A strong European influence was revealed in the gene pool of Creole fur sheep, which is, however, most likely caused by a later cross with merino sheep [84]. Whole-genome structural analysis of Spangler et al. showed the main influence of European breeds, especially Creole wool breeds [83]. Based on the mitochondrial maternal lineage, the origin cannot be specified because in the West African, European, and Canary sheep, maternal lineage B, which is also present in America, predominates [50]. However, another mitochondrial analysis found in Mexican Creole sheep several mitochondrial haplotypes common to both Creole sheep and two strains of Spanish sheep—Churro (Churra, Laxta, Churra Galega Mirandesa, Braganana) and Entrefino (Aragonesa, Manchega, Castellana, Castellana Stela) [81]. H2 haplotype was identified as the main ancestral mitochondrial haplotype in Mexican Creole sheep and haplotypes H21 and H32, present in both Cuba and Mexico as the possible original ones derived from hairy sheep [85].

However, it is already very difficult to study the evolution of “native” Creole sheep through genetics. The main reason is a strong disruption of the original gene pool by importing and crossing commercial breeds in the 19th and 20th centuries to Mexico [85,86,87] and other Central and South American countries [88,89].

The first sheep were brought to Australia from India, South Africa (thick-tailed), and Spain (merino) after 1788 and from the British Isles (Saxony Merino, Southdown, Romney) after 1840 [9]. It was, therefore, possible to assume the existence of the same lineages and most of the haplotypes (mt and Y) as in the populations from which Australian breeds originated. This expectation was confirmed by a study carried out on 18 breeds kept in Australia, which revealed 55% abundance of lineage B and 45% abundance of lineage A [69].

## 3. Haplogroups and Haplotypes of Male Y Chromosomes

The mutation rate of the male-specific region of the Y chromosome (MSY) is about fifty times lower than that of mtDNA, i.e., about 0.93 × 10^−10^ mutations per generation per site [35]. However, as with mitochondrial haplotypes, their use to study the phylogeny of a species is complicated by the fact that all members of the genus *Ovis* can interbreed and form fertile hybrids. Such insertion of a distant paternal or maternal lineage into a gene pool of another species often occurs in areas of overlap of distribution areas [13,18,29,64,90,91]. Estimates of urial and argali cleavage time have been performed in sheep based on male haplotypes so far only by [35]. In this case, the urial haplotype grouped with Asian mouflon haplotypes, in contrast to the mitochondrial genome where it formed a separate branch.

A key finding for the use of male haplotypes to study the population expansion of sheep populations was the discovery of eight SNP sites (*oY1*–*oY8*) in the sex-determining gene (SRY) on MSY [92]. And only one of them (*oY1*) showed variability even within the species and not only between them [93]. Subsequently, the microsatellite marker *SRYM18* was discovered and thanks to it, the first 18 Y chromosomal haplotypes H1–H18 were defined [93].

**Figure 4:**
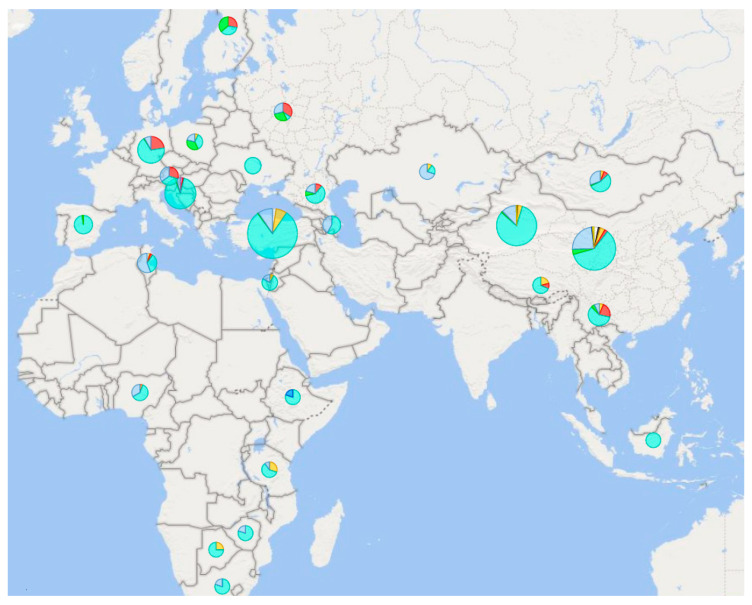
Species and frequencies of male haplotypes based on microsatellite markers in different areas of the Eastern Hemisphere. Color resolution of haplotypes: H4 H5 H6 H7 H8 H9 H10 H12 H18 H19 Ha Hb. The data are a compilation of the results of five studies [58,92,93,94,95,96]. On a scale of 5–386 samples per pie chart.

In general, not as many studies have been performed in the field of research on male sheep haplotypes as in the mitochondrial genome (Figure 4). The first large worldwide study revealed, with the help of two markers (SRY SNP *oY1* and microsatellite *SRYM18*) in domestic sheep, seven male haplotypes (H4–H10) that form two haplogroups [92]. Further research has taken over this methodological approach, including the nomenclature of the identified haplotypes. Follow-up studies revealed another H12 haplotype-specific for the Turkish Sakiz breed [93,94]. Other new haplotypes were subsequently discovered in Croatia—H18 [58] and northern China—H19, Ha, Hb [95,96]. Paternal genetic diversity of sheep has also been studied in Estonia and Finland on samples from the Bronze and Iron Ages [17]. However, only one SNP marker on the SRY gene (*G-oY1*) was monitored; thus, it does not provide any further information on the variability of the male genome in Europe [17].

**Figure 5:**
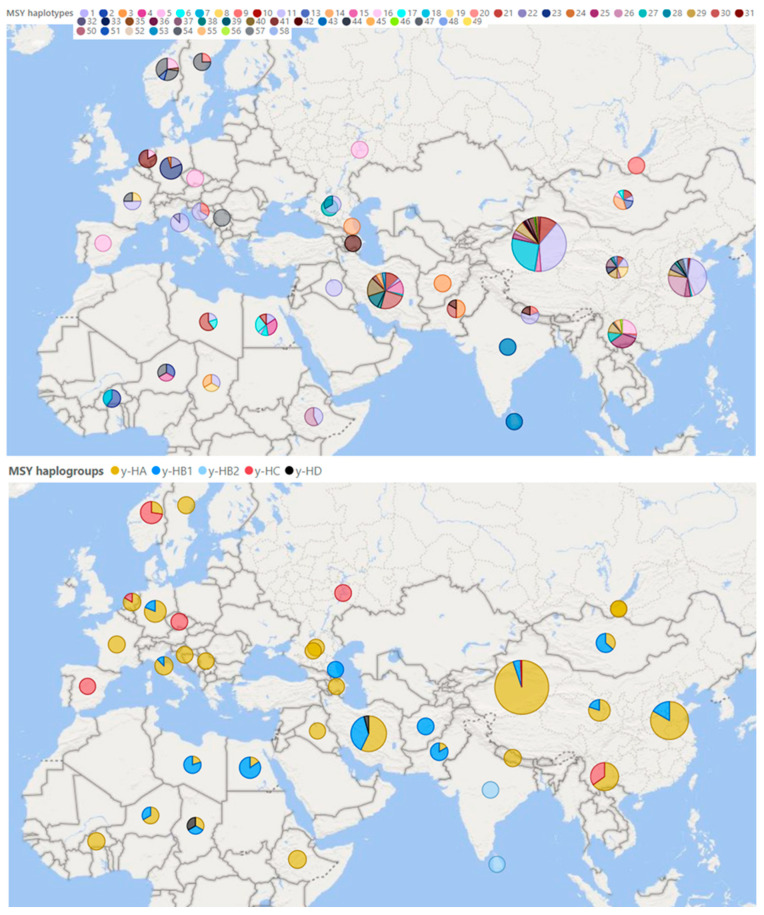
Male Y haplotypes and haplogroups occurring in native sheep breeds in the Eastern Hemisphere. Illustrated with program Power BI (Microsoft, 2022) [55]. The pictured data were taken over together with the nomenclature of the included haplotypes (H1–11; H13–33; H35–58) and haplogroups (y-HA; y-HB1; y-HB2; y-HC; y-HD) [35]. On a scale of 1–135 samples per pie chart.

The last breakthrough in this area was made in 2020 when whole-genome sequences were used to create a new set of MSY 495 SNPs in sheep [35]. Based on 179 samples of rams sequenced in the whole genome, they identified 49 different Y haplotypes. Based on a selection of 79 SNPs and two others published in previous studies (*oY1*; *oY2*), the study identified 58 other different haplotypes for domestic sheep belonging to four haplogroups: y-HA, y-HB, y-HC, and y- HD [35]. A total of 614 sheep from populations all over the world performed this genotyping. However, only native breeds were selected for our review (Figure 5).

Based on the genome-wide study of breeds from all over the world, a certain weak relationship was found between the degree of genetic variability and the distance from the domestication center [8]. For haplotypes inherited solely by paternal or maternal lineage, this phenomenon should be more pronounced because they are not affected by the recombination process and are transmitted from generation to generation in practically unchanged form. So far, however, current research does not suggest anything like this (Figure 4 and Figure 5).

Overall, the highest variability of male haplotypes is in sheep populations from areas close to the original center of domestication in the Middle East [95]. However, more data will be needed to support this assumption. Current findings so far point to the greatest variability in central China; see Figure 4. In contrast, the diversity of mitochondrial lines so far supports this assumption, as all known lines have been found in Turkey and Israel (Figure 3)—in the supposed original place of domestication.

## 4. Historical Background

It can be concluded that the highest diversity of the genome can be found as close as possible to the place of origin, as has been shown, for example, in humans [97]. For sheep, research in this area is a bit more complicated. Due to the controlled reproduction and trade of animals over long distances practically from the beginning of breeding, it is possible to infer a faster and more rapid spread of genetic material than was the case in humans. Initially, sheep farming focused mainly on meat, and specialization in secondary production elements, such as wool and milk, probably did not occur until many millennia later in Asia (7–6 thousand years BP) and millennia later in Europe [14,35,98]. Specialization in wool production probably originated in Southwest Asia and only then spread to Europe, which is confirmed by the study of retroviruses [14] and by the analysis of DNA of European sheep from the Bronze Age [99]. The introduction of a new breed into Central Europe in the late Stone Age is also indicated by archaeological findings. A comparison with older findings confirmed the increase in the body frame of sheep breeds bred in the area of Bohemia and Moravia [100] and since the beginning of the Bronze age as well in Hungary [101]. Another example is the spread of merino sheep from the Iberian Peninsula since the second half of the 15th century throughout Europe [102]. Most primitive breeds today have withstood the second wave of migration to Europe in a semi-wild or wild state in isolated areas without predators or outside areas economically prone to introgression [14].

The first expansion of sheep went along with man mainly overland to Europe, Africa, and then deeper into Asia during the Mesolithic and Neolithic periods. Sheep reached other continents (America and Australia) much later with the first European colonists. The use of haplotypes to study the distribution of domestic sheep and its breeds in modern history is almost impossible by modern modes of transport. Particularly, commercially used breeds create something like a “global population” in which it is not possible to exclude the genetic proximity of individuals on different continents. Mitochondrial and Y haplotypes do not generate sufficiently genetically unique markers to study genetic diversity at this level. However, their potential for studying the phylogeny of the species, and especially its population dispersion, remains untapped.

## 5. Conclusions

The aim of this study was to capture the process of monitoring the dispersion and development of domestic sheep populations in different parts of the world through the study of male and female non-recombinant sections of DNA. The current review supports the existence of one domestication center in the Middle East. Nevertheless, crossbreeding with wild sheep species has probably often happened and occasionally continues to occur even now. This could be the reason why central China shows such high variability in male haplotypes. However, it can also be caused by the extinction of these variants in the Middle East, with Central Asia being a kind of reservoir of variability originating from the ancient sheep brought in from the fertile crescent.

The main challenge for the future is to involve more countries and regions while increasing the number of animals used for sequencing. Only based on a larger amount of these data combined with the genetic material from excavations, it will be possible to identify other domestication centers or refute their existence. It would also be necessary to unify the methodology and nomenclature of haplotypes for better comparability of the results of different research. But perhaps we may never know the truth because a vast amount of information is already lost forever.

## Figures and Tables

**Table 1:** Comparison of approximated divergence times in million years ago (mya) of Argali, Urial and main mitochondrial haplogroup branches (A, B, D) between different studies.

Study	Data Origin	Times of Divergence in Mya		
		Argali	Urial	Branch of A, B, D haplogroups
Rezaei et al. (2010) [13]	Cytochrome b sequence	1.72	1.26	-
Meadows et al. (2011) [33]	Whole mitogenome	2.13	-	0.92
Lv et al. (2015) [34]	Whole mitogenome	2.93	2.60	0.89
Sanna et al. (2015) [32]	Whole mitogenome	1.11	0.89	0.30
Deng et al. (2020) [35]	Whole mitogenome	2.93	2.60	1.02

## Data Availability

Not applicable.
